# Association between iron deficiency anemia and risk of venous thromboembolism: a multi-institutional retrospective study

**DOI:** 10.3389/fnut.2025.1660622

**Published:** 2025-10-20

**Authors:** Kuo-Chuan Hung, Hsiu-Lan Weng, Chih-Wei Hsu, Yi-Chen Lai, I-Wen Chen

**Affiliations:** ^1^Department of Anesthesiology, Chi Mei Medical Center, Tainan, Taiwan; ^2^School of Medicine, College of Medicine, National Sun Yat-sen University, Kaohsiung, Taiwan; ^3^Department of Anesthesiology, E-Da Hospital, I-Shou University, Kaohsiung, Taiwan; ^4^Department of Psychiatry, Kaohsiung Chang Gung Memorial Hospital and Chang Gung University College of Medicine, Kaohsiung, Taiwan; ^5^Department of Anesthesiology, Chi Mei Medical Center, Liouying, Tainan, Taiwan

**Keywords:** iron deficiency anemia, venous thromboembolism, thrombocytosis, deep vein thrombosis, pulmonary embolism, retrospective cohort study

## Abstract

**Background:**

Iron deficiency anemia (IDA) represents the most prevalent nutritional deficiency globally, affecting approximately one-third of the world’s population. Despite its widespread occurrence, the association between IDA and venous thromboembolism (VTE) remains inadequately characterized. This study aimed to comprehensively evaluate the relationship between IDA and VTE risk using real-world clinical data.

**Methods:**

We conducted a multi-institutional retrospective cohort study utilizing the TriNetX platform to analyze electronic health records from 2010 to 2023. The study included 180,484 propensity-matched pairs, comparing patients with confirmed IDA to controls with dermatitis/eczema. Matching incorporated demographic, clinical, and laboratory variables to minimize confounding. The primary outcome was 1-year VTE risk, with secondary outcomes including all-cause mortality, intensive care unit (ICU) admission, upper extremity thrombosis, and thrombocytosis development. A mechanistic analysis excluded patients developing thrombocytosis to assess potential mediating pathways.

**Results:**

Patients with IDA demonstrated a 75% increased risk of VTE within 1 year compared to controls (hazard ratio [HR]: 1.75, 95% confidence interval [CI]: 1.58–1.94, *p* < 0.001). The association varied by VTE subtype, with pulmonary embolism showing a more pronounced relationship (HR 2.05, 95% CI: 1.76–2.38, *p* < 0.001) than deep vein thrombosis (HR 1.54, 95% CI: 1.35–1.75, *p* < 0.001). Risk was highest during the initial 3 months post-diagnosis (HR 2.09, 95% CI: 1.67–2.62, *p* < 0.001). When patients developing thrombocytosis were excluded, VTE risk was substantially attenuated (HR 1.19, 95% CI: 1.05–1.33, *p* = 0.004), suggesting reactive thrombocytosis mediates a significant portion of the excess risk. The thrombotic risk extended beyond traditional VTE sites, with upper extremity thrombosis occurring 2.5-fold more frequently (HR 2.50, 95% CI: 1.77–3.53, *p* < 0.001). IDA was also associated with increased mortality (HR 2.12, 95% CI: 1.93–2.32, *p* < 0.001) and ICU admission (HR 1.67, 95% CI: 1.52–1.83, *p* < 0.001).

**Conclusion:**

This large-scale study establishes IDA as a significant modifiable risk factor for VTE, with peak risk occurring early after diagnosis. The findings support enhanced clinical vigilance and consideration of prophylactic strategies for IDA patients, particularly during initial months following diagnosis.

## 1 Introduction

Venous thromboembolism (VTE), encompassing deep vein thrombosis (DVT) and pulmonary embolism (PE), represents a significant global health burden and is the third leading cause of cardiovascular death worldwide (1, 2). In Western populations, VTE and PE occur at estimated rates of approximately 0.87 to 1.82 and 0.45 to 0.95 per 1,000 person-years, respectively (3–6). In the United States alone, this corresponds to an annual burden of 300,000 to 600,000 VTE cases, with associated healthcare costs exceeding $7 billion annually (7). The pathophysiology of VTE involves the classical Virchow’s triad of endothelial injury, blood stasis, and hypercoagulability (8–10). Beyond its acute morbidity and mortality, VTE imposes substantial long-term complications, including post-thrombotic syndrome, chronic thromboembolic pulmonary hypertension, and increased risk of recurrence, contributing to reduced quality of life and increased healthcare costs (11–14).

Iron deficiency anemia (IDA) is the most prevalent nutritional deficiency globally, affecting approximately one-third of the world’s population (15, 16). Although traditionally viewed as a benign hematologic condition, emerging evidence suggests that IDA may contribute to thrombotic complications through several mechanisms. Iron deficiency can induce reactive thrombocytosis (17, 18), leading to a hypercoagulable state, and may cause decreased antioxidant defense, resulting in increased oxidant stress and platelet aggregation (19–21). In addition, increased red cell distribution width (RDW) associated with IDA can disturb laminar blood flow and increase blood viscosity, thereby promoting thrombotic risk (22, 23). However, the existing literature supporting the association between IDA and VTE predominantly consists of isolated case reports (24–27), which have been insufficient to establish definitive evidence. A population-based case–control study on 2,522 patients with VTE reported that individuals with prior IDA had a 43% higher odds of developing VTE than those without IDA (28). However, the study’s reliance on basic demographic matching may limit causal inference, and it did not address the temporal relationship between IDA and VTE or the potential mediating role of thrombocytosis (28).

We hypothesized that patients with IDA have an increased risk of developing VTE compared to matched controls, potentially mediated through reactive thrombocytosis. The primary aim of this multi-institutional retrospective cohort study was to evaluate the association between IDA and the risk of VTE using a large, real-world clinical database.

## 2 Methods

### 2.1 Data sources and ethical statement

We conducted a multi-institutional, retrospective, matched cohort study to evaluate the association between IDA and the risk of VTE, including DVT and PE. This retrospective cohort study was conducted using the TriNetX platform, a global health research network that compiles de-identified electronic health record data from participating healthcare institutions across several countries (29, 30). The database includes a wide range of real-world clinical information, such as patient demographics, diagnoses, procedures, medications, and laboratory values. The reliability and validity of the TriNetX research platform are supported by numerous published studies using its data for large-scale clinical investigations (31–34). The study protocol followed the ethical principles of the Declaration of Helsinki and was approved by the Institutional Review Board of Chi Mei Medical Center (IRB number: 11403-E01). As the study used anonymized data from an existing database, the need for informed consent was waived in accordance with the relevant ethical guidelines.

### 2.2 Inclusion criteria

The study population included adult patients (aged ≥18 years) with electronic health records from participating healthcare institutions between January 2010 and December 2023. Patients were eligible for inclusion in the IDA group if they had a first documented diagnosis of IDA (ICD-10 codes D50) during the study period (2010–2023). To improve diagnostic specificity and ensure chronicity, only patients with a second recorded diagnosis of IDA between 3 and 12 months after the initial diagnosis were included. The index date for the IDA group was defined as the date of the first IDA diagnosis. The control group included patients who had their first documented diagnosis of dermatitis or eczema (ICD-10 codes L20–L23) during the study period. The index date was defined as the date of the first qualifying dermatitis/eczema diagnosis. Dermatitis/eczema was selected as the control condition to ensure comparable healthcare utilization while minimizing confounding from conditions known to influence thrombotic risk.

### 2.3 Exclusion criteria

Patients in the IDA group were excluded if they had any other type of anemia (e.g., vitamin B12 deficiency, folate deficiency, hemolytic anemia, aplastic anemia, or anemia related to chronic kidney disease) recorded at any time from before the index date to the follow-up period. Similarly, patients in the control group were excluded if they were diagnosed with anemia within the same timeframe.

Both groups were excluded if they had a prior diagnosis of any venous thrombosis (ICD-10 code I82) or PE (ICD-10 code I26) before the start of follow-up. Additional exclusion criteria for both groups included a history of end-stage renal disease, splenectomy, immune thrombocytopenia, secondary thrombocytopenia, polycythemia vera, essential thrombocythemia, hemiplegia, hemiparesis, paraplegia, or quadriplegia, as well as the use of hormone replacement therapy or oral contraceptives documented at any time from before the index date through the follow-up period. Patients were also excluded if they had a documented pregnancy, lower extremity surgery, cerebral infarction, or intracerebral hemorrhage during the follow-up period. These exclusions were implemented to minimize confounding from the established VTE risk factors.

### 2.4 Outcomes and follow-up

The primary outcome was the development of VTE (i.e., DVT or PE). Secondary outcomes included risks of all-cause mortality, thrombosis of the upper extremity, intensive care unit (ICU) admission, and thrombocytosis (≥450,000/μL). The follow-up period was defined as 1–12 months following the index date. A 1-month washout period after the index date aimed to reduce the misclassification of pre-existing VTE events and to better reflect the incident risk attributable to IDA.

### 2.5 Data collection

The baseline demographic and clinical characteristics of all eligible patients were extracted from the TriNetX database. Demographic variables included age at the index date, sex, race/ethnicity, and body mass index (BMI). Clinical comorbidities were identified using the International Classification of Diseases, Tenth Revision (ICD-10) diagnostic codes and included essential hypertension, disorders of lipoprotein metabolism, overweight and obesity, diabetes mellitus, neoplasms, ischemic heart diseases, nicotine dependence, diseases of the liver, chronic kidney disease, heart failure, COVID-19, cerebrovascular diseases, systemic connective tissue disorders, rheumatoid arthritis, alcohol-related disorders, gout, varicose veins of the lower extremities, systemic lupus erythematosus, and malnutrition. The laboratory parameters collected included platelet count, hemoglobin A1c levels, serum albumin levels, and estimated glomerular filtration rate (eGFR). Medication use, including platelet aggregation inhibitors, anticoagulants, and iron preparations, was recorded.

### 2.6 Additional analysis (mechanism of VTE)

To assess whether IDA-induced thrombocytosis contributes to the development of VTE (17, 18), we conducted an additional mechanistic analysis. In this analysis, we excluded patients who developed thrombocytosis (platelet count ≥ 450 × 10^3^/μL) during the follow-up period and re-examined whether IDA remained independently associated with VTE development. This approach aimed to isolate the direct thrombotic effects of IDA from those potentially mediated by reactive thrombocytosis. This analysis used the same statistical methodology as the primary analysis, including Cox proportional hazards regression, with the same follow-up periods and outcome definitions.

### 2.7 Subgroup analyses

Pre-specified subgroup analyses were performed to assess whether patient characteristics or other factors modified the association between IDA and VTE risk. Subgroups were defined by sex (male vs. female), age categories (>65 years vs. 18–65 years), diabetes mellitus status (present vs. absent), nicotine dependence (present vs. absent), BMI (≥30 and <30 kg/m^2^), and study period (2010–2019 vs. 2020–2023).

### 2.8 Statistical analysis

Continuous variables are presented as mean ± standard deviation, while categorical variables are expressed as frequencies and percentages. To address potential selection bias, 1:1 propensity score matching was performed using a caliper width of 0.1 standard deviations of the logit of the propensity score. The propensity score model included all baseline demographic characteristics, comorbidities, laboratory parameters, and medication use. Matching was conducted without replacement using a greedy nearest-neighbor algorithm. Baseline characteristics between the IDA and control groups were compared using standardized mean differences (SMD), with SMD values <0.1 indicating adequate balance between groups.

Time-to-event analyses were performed using Cox proportional hazards regression models to estimate hazard ratios (HR) and 95% confidence intervals (CI) for the association between IDA and study outcomes. The proportional hazard assumption was assessed using Schoenfeld residuals. To account for multiple comparisons, Bonferroni correction was applied. For all analyses, statistical significance was set at *p* < 0.01 (0.05/5 outcomes). All statistical analyses were performed using the built-in analytical tools of the TriNetX platform. Two-sided *p*-values have been reported throughout the manuscript.

## 3 Results

### 3.1 Patient selection and baseline characteristics

**Figure 1:**
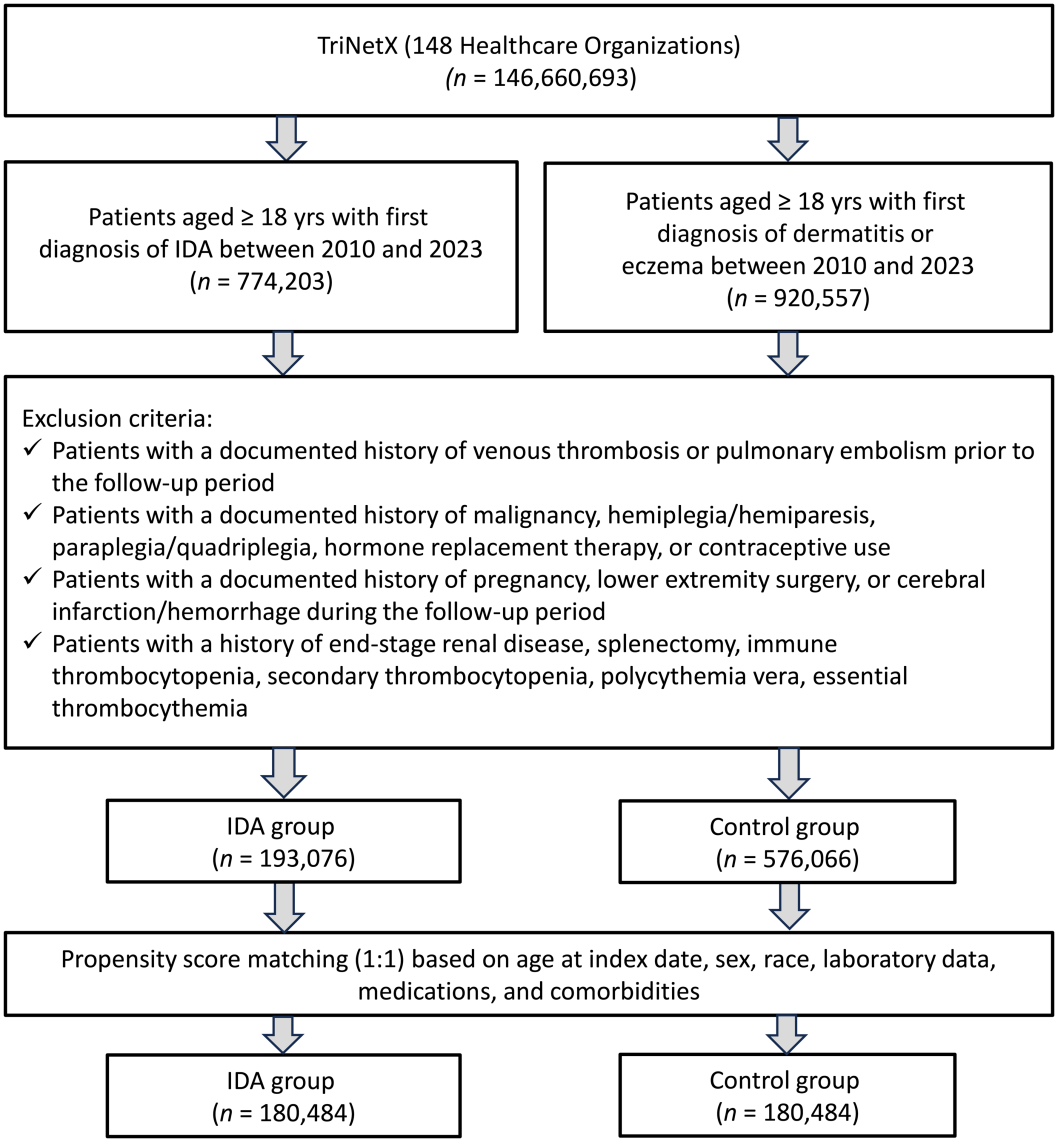
Patient selection from the TriNetx database. IDA, iron deficiency anemia.

The initial cohort comprised 193,076 patients with IDA and 576,066 control patients with dermatitis or eczema. After propensity score matching, each group included 180,484 patients (Figure 1). The matched cohort demonstrated excellent balance across all baseline characteristics, with SMD consistently below 0.1 (Table 1). Patients in both groups had a mean age of approximately 49 years, with females comprising 75.8% of the IDA group and 77.5% of the control group. The most prevalent comorbidities included essential hypertension (21.8% vs. 22.3%), disorders of lipoprotein metabolism (17.8% vs. 18.3%), and overweight/obesity (11.9% vs. 12.0%) in the IDA and control groups, respectively. Notably, baseline thrombocytosis (platelet count >450 × 10^3^/μL) was present in 2.7% of IDA patients compared to 2.1% of controls. Medication profiles, including the use of iron preparations (5.6% vs. 4.7%), were well balanced between the groups.

**Table 1:** Baseline characteristics of patients before and after propensity score matching.

Variables	Before matching		After matching			
	IDA group (*n* = 193,076)	Control group (*n* = 576,066)	SMD†	IDA group (*n* = 180,484)	Control group (*n* = 180,484)	SMD†
Patient characteristics						
Age at index (years)	48.6 ± 19.3	43.6 ± 21.5	0.243	48.7 ± 19.3	49.4 ± 19.7	0.040
BMI (kg/m^2^)	30.6 ± 8.7	28.8 ± 7.5	0.215	30.4 ± 8.6	29.9 ± 8.0	0.066
Female (n, %)	148,245 (76.8)	337,612 (58.6)	0.396	136,809 (75.8)	139,934 (77.5)	0.041
White (n, %)	101,091 (52.4)	317,826 (55.2)	0.056	95,352 (52.8)	95,791 (53.1)	0.005
Unknown Race (n, %)	37,846 (19.6)	104,362 (18.1)	0.038	35,879 (19.9)	35,792 (19.8)	0.001
Black or African American (n, %)	34,007 (17.6)	76,399 (13.3)	0.121	30,159 (16.7)	29,396 (16.3)	0.011
Asian (n, %)	10,235 (5.3)	48,367 (8.4)	0.123	9,944 (5.5)	10,276 (5.7)	0.008
Other Race (n, %)	7,561 (3.9)	21,075 (3.7)	0.013	6,927 (3.8)	6,887 (3.8)	0.001
Comorbidities						
Essential (primary) hypertension (n, %)	43,131 (22.3)	104,227 (18.1)	0.106	39,255 (21.8)	40,223 (22.3)	0.013
Disorders of lipoprotein metabolism and other lipidemias (n, %)	34,577 (17.9)	99,837 (17.3)	0.015	32,147 (17.8)	33,021 (18.3)	0.013
Overweight and obesity (n, %)	24,694 (12.8)	49,429 (8.6)	0.137	21,545 (11.9)	21,678 (12.0)	0.002
Diabetes mellitus (n, %)	22,819 (11.8)	44,779 (7.8)	0.136	20,321 (11.3)	20,469 (11.3)	0.003
Neoplasms (n, %)	16,784 (8.7)	57,316 (10.0)	0.043	15,659 (8.7)	15,673 (8.7)	<0.001
Ischemic heart diseases (n, %)	10,094 (5.2)	19,112 (3.3)	0.095	8,976 (5.0)	8,775 (4.9)	0.005
Nicotine dependence (n, %)	7,524 (3.9)	24,474 (4.2)	0.018	6,928 (3.8)	7,100 (3.9)	0.005
Diseases of liver (n, %)	6,091 (3.2)	14,184 (2.5)	0.042	5,548 (3.1)	5,462 (3.0)	0.003
Chronic kidney disease (CKD) (n, %)	6,416 (3.3)	9,338 (1.6)	0.110	5,502 (3.0)	5,153 (2.9)	0.011
Heart failure (n, %)	5,246 (2.7)	6,358 (1.1)	0.118	4,312 (2.4)	3,963 (2.2)	0.013
COVID-19 (n, %)	4,121 (2.1)	8,791 (1.5)	0.045	3,658 (2.0)	3,638 (2.0)	0.001
Cerebrovascular diseases (n, %)	3,604 (1.9)	8,128 (1.4)	0.036	3,258 (1.8)	3,112 (1.7)	0.006
Systemic connective tissue disorders (n, %)	3,544 (1.8)	8,007 (1.4)	0.035	3,227 (1.8)	3,338 (1.8)	0.005
Other rheumatoid arthritis (n, %)	2,460 (1.3)	5,326 (0.9)	0.034	2,221 (1.2)	2,299 (1.3)	0.004
Alcohol related disorders (n, %)	1975 (1.0)	6,147 (1.1)	0.004	1807 (1.0)	1838 (1.0)	0.002
Gout (n, %)	1851 (1.0)	6,475 (1.1)	0.016	1730 (1.0)	1,693 (0.9)	0.002
Varicose veins of lower extremities (n, %)	1,507 (0.8)	5,217 (0.9)	0.014	1,424 (0.8)	1,499 (0.8)	0.005
Long term use of steroids (n, %)	1,534 (0.8)	3,137 (0.5)	0.031	1,308 (0.7)	1,321 (0.7)	0.001
Systemic lupus erythematosus (n, %)	1,174 (0.6)	2,320 (0.4)	0.029	1,064 (0.6)	1,098 (0.6)	0.002
Malnutrition (n, %)	997 (0.5)	907 (0.2)	0.062	714 (0.4)	635 (0.4)	0.007
Laboratory data						
HbA1c ≥ 7% (n, %)	10,482 (5.4)	21,257 (3.7)	0.083	9,235 (5.1)	9,065 (5.0)	0.004
Albumin g/dL (≥3.5 g/dL) (n, %)	71,907 (37.2)	156,744 (27.2)	0.216	63,865 (35.4)	65,303 (36.2)	0.017
eGFR>60 mL/min/1.73 m^2^ (n, %)	75,362 (39.0)	171,952 (29.8)	0.194	67,192 (37.2)	69,261 (38.4)	0.024
Platelet > 450 × 10^3^/μL (n, %)	8,960 (4.6)	4,152 (0.7)	0.244	4,853 (2.7)	3,828 (2.1)	0.037
Platelet 100–450 × 10^3^/μL (n, %)	88,141 (45.7)	178,983 (31.1)	0.303	78,490 (43.5)	81,085 (44.9)	0.029
Platelet< 100 × 10^3^/μL (n, %)	1,411 (0.7)	2084 (0.4)	0.050	1,194 (0.7)	1,155 (0.6)	0.003
Medications						
Platelet aggregation inhibitors (n, %)	13,585 (7.0)	29,478 (5.1)	0.080	11,793 (6.5)	11,533 (6.4)	0.006
Anticoagulants (n, %)	13,476 (7.0)	20,703 (3.6)	0.152	10,999 (6.1)	10,546 (5.8)	0.011
Iron preparations (n, %)	19,314 (10.0)	8,749 (1.5)	0.370	10,073 (5.6)	8,400 (4.7)	0.042

IDA, iron deficiency anemia; BMI, body mass index; SMD, standardized mean differences; †SMD values <0.1 indicate adequate balance between groups; eGFR, estimated Glomerular Filtration Rate.

### 3.2 Outcomes

#### 3.2.1 Risk of VTE at 1-year follow-up

During the 1-year follow-up, patients with IDA demonstrated a substantially higher risk of VTE than controls (HR 1.75, 95% CI: 1.58–1.94, *p* < 0.001; Table 2). This elevated risk manifested differently across VTE subtypes: DVT increased by 54% (HR 1.54, 95% CI: 1.35–1.75, *p* < 0.001), while PE showed an even more pronounced association (HR 2.05, 95% CI: 1.76–2.38, *p* < 0.001).

**Table 2:** Association between iron deficiency anemia and 12-month outcomes.

Outcomes	IDA group (*n* = 180,484)	Control group (*n* = 180,484)	HR (95% CI)	Log-rank test: *p*-value*
	Events (%)	Events (%)		
VTE†	1,002 (0.56)	575 (0.32)	1.75 (1.58–1.94)	< 0.001
DVT	578 (0.32)	378 (0.21)	1.54 (1.35–1.75)	< 0.001
Pulmonary embolism	511 (0.28)	251 (0.14)	2.05 (1.76–2.38)	< 0.001
Thrombosis of upper extremity	112 (0.06)	45 (0.03)	2.5 (1.77–3.53)	< 0.001
Mortality	1,367 (0.76)	651 (0.36)	2.12 (1.93–2.32)	< 0.001
ICU admission	1,166 (0.65)	704 (0.39)	1.67 (1.52–1.83)	< 0.001
Thrombocytosis (Platelet > 450 × 10^3^/μL)	6,828 (3.78)	1911 (1.06)	3.64 (3.46–3.83)	< 0.001

IDA, Iron deficiency anemia; VTE, venous thromboembolism; †VTE included acute deep vein thrombosis and pulmonary embolism; DVT, deep vein thrombosis; ICU, intensive care unit; HR, hazard ratio; CI, confidence interval. *p < 0.0125 was considered significance following Bonferroni correction.

The thrombotic risk extended beyond VTE, with upper extremity thrombosis occurring 2.5-fold more frequently in IDA patients (HR 2.50, 95% CI: 1.77–3.53, *p* < 0.001). Additionally, patients faced a nearly fourfold increased risk of developing thrombocytosis (HR 3.64, 95% CI: 3.46–3.83, *p* < 0.001). These vascular complications translate into severe clinical consequences. All-cause mortality increased significantly in the IDA group (HR 2.12, 95% CI: 1.93–2.32, *p* < 0.001), accompanied by increased ICU admissions (HR 1.67, 95% CI: 1.52–1.83, *p* < 0.001).

#### 3.2.2 Risk of VTE at 3-month follow-up

Short-term analysis revealed a stronger association between IDA (IDA) and the risk of VTE during the initial 3 months of follow-up (Table 3). Patients with IDA exhibited more than twice the risk of developing VTE compared to controls (HR 2.09, 95% CI: 1.67–2.62, *p* < 0.001), representing a notably higher risk magnitude than the 1-year analysis (HR 1.75). This elevated risk was consistent across VTE subtypes: DVT (HR 2.04, 95% CI: 1.54–2.71, *p* < 0.001) and PE (HR 2.08, 95% CI: 1.49–2.89, *p* < 0.001) both showed significant increases in the IDA group. Notably, while DVT risk remained substantially elevated compared to the 1-year estimate (HR 1.54), PE risk was comparable between the short-term and long-term follow-up periods.

**Table 3:** Association between iron deficiency anemia and 3-month outcomes.

Outcomes	IDA group (*n* = 180,484)	Control group (*n* = 180,484)	HR (95% CI)	Log-rank test: *p*-value*
	Events (%)	Events (%)		
VTE†	234 (0.13)	112 (0.06)	2.09 (1.67–2.62)	< 0.001
DVT	145 (0.08)	71 (0.04)	2.04 (1.54–2.71)	< 0.001
Pulmonary embolism	108 (0.06)	52 (0.03)	2.08 (1.49–2.89)	< 0.001
Thrombosis of upper extremity	25 (0.014)	10 (0.006)	3.13 (1.41–6.93)	0.003
Mortality	39 (0.02)	13 (0.01)	3.00 (1.60–5.62)	< 0.001
ICU admission	191 (0.11)	131 (0.07)	1.46 (1.17–1.82)	< 0.001
Thrombocytosis (Platelet > 450 × 10^3^/μL)	1967 (1.09)	481 (0.27)	4.11 (3.72–4.54)	< 0.001

IDA, Iron deficiency anemia; VTE, venous thromboembolism; †VTE included acute deep vein thrombosis and pulmonary embolism; DVT, deep vein thrombosis; ICU, intensive care unit; HR, hazard ratio; CI, confidence interval. *p < 0.01 was considered significance following Bonferroni correction.

The most pronounced disparity was observed in the risks of upper extremity thrombosis (HR 3.13, 95% CI: 1.41–6.93, *p* = 0.003) and early mortality (HR 3.00, 95% CI: 1.60–5.62, *p* < 0.001), with a threefold higher risk among IDA patients compared to controls. Similarly, the onset of thrombocytosis was markedly accelerated within the same period, with IDA patients demonstrating a fourfold increased risk (HR 4.11, 95% CI: 3.72–4.54, *p* < 0.001) that surpassed the 1-year thrombocytosis risk (HR 3.64). These findings suggest that the period of highest risk for adverse outcomes occurs within the initial months after IDA diagnosis.

### 3.3 Analysis after excluding patients with thrombocytosis

To assess whether reactive thrombocytosis mediates the increased risk of VTE associated with IDA, we performed an additional analysis excluding patients who developed thrombocytosis during follow-up (Table 4). The analysis included 110,342 patients in each group. Although the association between IDA and VTE remained statistically significant, it was notably attenuated (HR 1.19, 95% CI: 1.05–1.33, *p* = 0.004). When VTE components were evaluated separately, the association with DVT was no longer significant (HR 1.06, 95% CI: 0.92–1.23, *p* = 0.404), whereas PE retained a modest but statistically significant association (HR 1.28, 95% CI: 1.08–1.53, *p* = 0.005). These findings suggest that thrombocytosis may play a more prominent role in the development of DVT than PE in patients with IDA.

**Table 4:** Association between iron deficiency anemia and 1-year outcomes after excluding patients with thrombocytosis during the follow-up.

Outcomes	IDA group (*n* = 110,342)	Control group (*n* = 110,342)	HR (95% CI)	Log-rank test: *p*-value*
	Events (%)	Events (%)		
VTE†	607 (0.55)	517 (0.47)	1.19 (1.05–1.33)	0.004
DVT	373 (0.34)	354 (0.32)	1.06 (0.92–1.23)	0.404
Pulmonary embolism	281 (0.26)	221 (0.20)	1.28 (1.08–1.53)	0.005
Thrombosis of upper extremity	71 (0.06)	50 (0.05)	1.43 (0.99–2.06)	0.050
Mortality	609 (0.55)	320 (0.29)	1.93 (1.69–2.21)	< 0.001
ICU admission	841 (0.76)	779 (0.71)	1.09 (1.17–1.82)	0.080
Thrombocytosis (Platelet > 450 × 10^3^/μL)	-	-	-	-

IDA, Iron deficiency anemia; VTE, venous thromboembolism; †VTE included acute deep vein thrombosis and pulmonary embolism; DVT, deep vein thrombosis; ICU, intensive care unit; HR, hazard ratio; CI, confidence interval. *p < 0.01 was considered significance following Bonferroni correction.

Importantly, the association between IDA and all-cause mortality persisted even after excluding patients with thrombocytosis (HR 1.93, 95% CI: 1.69–2.21, *p* < 0.001), while the risk of ICU admission was reduced and no longer statistically significant after Bonferroni correction (HR 1.09, 95% CI: 1.17–1.82, *p* = 0.080). These results imply that the elevated 12-month mortality risk in IDA is likely independent of thrombocytosis or VTE, whereas ICU admission may be partially driven by thrombotic complications.

### 3.4 Subgroup analyses

Subgroup analyses showed a consistent increase in VTE risk associated with IDA across all patient characteristics (Table 5). The association remained significant in both males (HR 1.67, 95% CI: 1.37–2.04) and females (HR 1.88, 95% CI: 1.66–2.14), with no interaction by sex (*p* = 0.318). Risk elevation was observed in both age groups, with a slightly stronger effect observed in the younger patients. In addition, BMI, nicotine dependence, and diabetes did not modify this association. The association between IDA and risk of VTE was also consistent across study periods, with similar risks for 2010–2019 (HR 1.80) and 2020–2023 (HR 1.68). No significant interactions were detected, indicating that the VTE risk linked to IDA is robust across subgroups.

**Table 5:** Subgroup analysis of association between iron deficiency anemia and risk of VTE at 1-year follow-up.

Subgroup analysis	Total number	HR (95% CI)	*p*-value*	P for interaction
Sex				
Male	70,398	1.67 (1.37–2.04)	<0.001	reference
Female	273,700	1.88 (1.66–2.14)	<0.001	0.318
Age				
18–60 years	215,786	1.97 (1.67–2.32)	<0.001	reference
>60 years	136,694	1.60 (1.40–1.83)	<0.001	0.063
Body mass index				
<30 kg/m^2^	292,690	1.90 (1.69–2.15)	<0.001	reference
≥30 kg/m^2^	67,602	1.61 (1.32–1.98)	<0.001	0.158
Nicotine dependence				
Yes	10,768	1.94 (1.21–3.12)	0.005	reference
No	349,896	1.72 (1.55–1.91)	<0.001	0.657
Diabetes Mellitus				
Yes	37,500	1.47 (1.15–1.89)	0.002	reference
No	322,758	1.90 (1.69–2.13)	<0.001	0.05
Study year				
2010–2019	199,840	1.80 (1.57–2.05)	<0.001	reference
2020–2023	249,076	1.68 (1.48–1.91)	<0.001	0.465

HR, hazard ratio; CI, confidence interval; *p < 0.01 was considered significance following Bonferroni correction.

## 4 Discussion

Our analysis of 180,484 propensity-matched pairs demonstrated that patients with IDA face a 75% higher risk of developing VTE within 1 year compared to controls. The association was particularly pronounced for PE, which showed a more than two-fold increased risk, while DVT exhibited a modest but still significant 54% increase in risk. The thrombotic risk was most pronounced during the initial 3 months following IDA diagnosis, with patients experiencing more than double the risk of VTE during this critical early period. Importantly, when patients who developed thrombocytosis were excluded from the analysis, the VTE association was substantially attenuated (HR 1.19), suggesting that reactive thrombocytosis mediates a significant portion of the IDA-associated thrombotic risk. Additionally, IDA is associated with substantially increased risks of all-cause mortality, ICU admission, and the development of reactive thrombocytosis, highlighting the broader clinical implications of this nutritional deficiency beyond its hematologic manifestations.

Despite the clinical importance of understanding the relationship between iron-deficiency anemia and thrombosis, the evidence base remains inadequate. Hung et al. conducted a population-based case–control study (28) that identified a 43% increase in VTE odds among patients with prior IDA. However, this investigation was fundamentally limited by rudimentary demographic matching procedures that failed to account for critical confounding variables. Perhaps more significantly, the study design could not establish a crucial temporal sequence between IDA onset and subsequent thrombotic events nor did it explore the potential mechanistic role of reactive thrombocytosis in mediating this relationship. Another large-scale study by Song et al. (35) demonstrated important findings—including a 32.6% rate of thrombocytosis in IDA patients and a 2-fold increased thrombotic risk in those with thrombocytosis compared to IDA patients without thrombocytosis—it had several important limitations. The study was limited to a single healthcare system, potentially affecting generalizability (35). More importantly, the study design did not directly compare patients with IDA to healthy controls without iron deficiency, making it impossible to quantify the absolute thrombotic risk attributable to IDA itself. Additionally, the lack of systematic propensity matching may have artificially elevated the overall thrombotic rates and limited the ability to control for important confounding variables.

Our study enhances the current understanding of the relationship between IDA and VTE by employing a prospective analytic framework with confounder adjustment. While previous retrospective studies, such as the Taiwanese case–control analysis (28), suggested a potential association, our findings quantify this risk using time-to-event analysis, offering greater clinical applicability. The observed elevated VTE risk highlights the importance of recognizing IDA as a potentially modifiable risk factor in thrombotic disease prevention and underscores the value of precise risk estimation for guiding individualized patient management. The novelty of our study lies in several key methodological advances over previous studies (28, 35). First, we employed rigorous propensity score matching across a comprehensive set of demographic, clinical, and laboratory variables to ensure balanced comparison groups and minimize selection bias. Second, we established clear temporal relationships by implementing a one-month washout period and following patients prospectively for VTE development. Third, our analysis included detailed laboratory parameters and medication exposures that were absent from previous studies (28, 35). Most importantly, we conducted mechanistic analyses to evaluate the role of reactive thrombocytosis in mediating the IDA-VTE relationship, thereby providing novel insights into the pathophysiological pathways underlying this association.

Our study revealed intriguing differences in how IDA affects the risk of specific VTE subtypes, with important implications for understanding the underlying pathophysiology. The more pronounced association with PE compared to DVT suggests that IDA may differentially impact arterial versus venous thrombotic mechanisms. This finding contrasts with the Taiwanese study (28), which reported significant associations with DVT but not with PE, highlighting the importance of adequate sample sizes and rigorous methodology in detecting these associations. Temporal analysis provides additional insights into risk patterns over time. During the short-term follow-up period of one–three months, the risk elevation for both DVT and PE was remarkably similar and pronounced, suggesting that the acute phase following IDA diagnosis represents a period of particularly heightened thrombotic risk. However, over the extended 12-month follow-up period, the DVT risk showed relative attenuation, while the PE risk remained substantially elevated. This temporal divergence may reflect different pathophysiological mechanisms, with reactive thrombocytosis potentially playing a more prominent role in DVT development during the acute phase, while other IDA-related factors, such as hypoxia-induced endothelial changes or altered blood rheology, may have more sustained effects on pulmonary circulation. These differential risk patterns also suggest potential clinical implications for monitoring and prophylaxis strategies. The particularly elevated risk during the initial months following IDA diagnosis argues for heightened clinical vigilance during this period, whereas the sustained elevation in PE risk throughout the follow-up period suggests that the prothrombotic effects of IDA may persist even as the acute phase resolves.

Our mechanistic analysis, which excluded patients who developed thrombocytosis during follow-up, provides insights into the relative contributions of thrombocytosis-dependent and thrombocytosis-independent pathways. The substantial attenuation of the overall VTE association after excluding patients with thrombocytosis, with the hazard ratio decreasing from 1.75 to 1.19, suggests that reactive thrombocytosis mediates approximately one-third of the excess VTE risk associated with IDA. More strikingly, the DVT risk became non-significant after excluding thrombocytic patients, while the PE risk remained elevated, suggesting fundamental differences in the pathophysiological mechanisms underlying these two manifestations of VTE. The differential effects on DVT versus PE suggest that while reactive thrombocytosis may be the primary driver of venous thrombosis in the peripheral circulation, PE may result from additional mechanisms such as altered pulmonary vascular reactivity, endothelial dysfunction, or changes in blood rheology that persist independent of platelet count elevation. This mechanistic understanding has important clinical implications, suggesting that monitoring platelet counts in IDA patients may help identify those at the highest risk for DVT, while all IDA patients may remain at elevated risk for PE regardless of platelet count. Furthermore, these insights may inform future research into targeted prevention strategies, with antiplatelet therapy potentially being more effective for DVT prevention, while anticoagulation may be necessary for comprehensive VTE prophylaxis in high-risk IDA patients.

Beyond reactive thrombocytosis, several additional mechanisms may contribute to the prothrombotic risk associated with iron deficiency anemia. Hypoxia-induced endothelial dysfunction can impair vascular integrity and promote thrombogenesis (36, 37). Elevated red cell distribution width (RDW) and increased blood viscosity, both recognized sequelae of iron deficiency, are also associated with disturbed blood flow and a heightened risk of VTE (21–23). Furthermore, iron deficiency is linked to increased oxidative stress and low-grade inflammation, which can lead to platelet hyperactivation and further enhance the prothrombotic milieu (19, 21). Consideration of these diverse thrombocytosis-independent pathways offers a more comprehensive understanding of the multifactorial relationship between IDA and VTE risk.

In the current study, this elevated risk of mortality persisted even after excluding patients who developed thrombocytosis, indicating that the increased death risk is largely independent of reactive thrombocytosis and VTE development. This finding suggests that IDA serves as a marker of overall physiological vulnerability rather than simply predisposing to specific thrombotic complications. The increased ICU admission rate in patients with IDA provides further evidence of the broader clinical impact of this condition. The attenuation of ICU admission risk after excluding patients with thrombocytosis suggests that a substantial portion of the acute care needs in IDA patients may be related to thrombotic complications. This finding has important implications for healthcare resources and supports the potential value of early identification and management of reactive thrombocytosis in patients with IDA to prevent severe complications requiring intensive care. The results support aggressive investigation of the underlying causes of IDA and consideration of more intensive monitoring strategies for patients with severe or persistent iron deficiency.

The selection of patients with dermatitis or eczema as the control group was based on the need to ensure comparable healthcare utilization patterns and to minimize confounding from conditions directly related to thrombotic risk. Dermatologic diseases such as dermatitis or eczema are common, typically benign, and not independently associated with increased risk of VTE, making them a pragmatic comparator for this real-world analysis. However, we acknowledge that the use of this control group may introduce residual confounding, as unmeasured factors unique to patients with dermatitis or eczema (e.g., chronic inflammation or medication exposure) could influence VTE risk. Additionally, certain demographic or behavioral characteristics may differ between groups despite propensity score matching. Future studies should consider sensitivity analyses using alternative control groups, such as matched healthy individuals or patients with other non-thrombotic chronic conditions, to further validate the observed associations and assess the robustness of these findings. This approach would help clarify the extent to which the observed associations are attributable to IDA itself rather than potential characteristics of the comparator group.

In our analysis, the incidence of upper extremity thrombosis ranged from 0.03 to 0.06%, which closely aligns with previously reported estimates in the general population. For example, Oymak et al. (38) documented a prevalence of 0.04% among adults in a large hospital-based cohort, with most cases linked to malignancy, chronic illness, or catheter placement. These findings suggest that the frequency of upper extremity thrombosis observed in our study is consistent with expected background rates rather than an overestimation. Importantly, while upper extremity thrombosis is typically associated with local precipitating factors such as venous catheters or mechanical compression, our data indicate that patients with IDA may also be susceptible to thrombotic complications beyond the lower extremities. This observation reinforces the need for vigilance in clinical monitoring, although secondary risk factors likely remain central to UET pathogenesis.

Several important limitations must be acknowledged when interpreting our findings. First, as a retrospective observational study using electronic health record data, we could not establish definitive causal relationships between IDA and VTE despite the temporal sequencing and matching procedures employed. Second, our reliance on ICD-10 diagnostic codes for both exposure and outcome ascertainment introduces the potential for misclassification bias. However, we implemented several strategies to minimize this limitation, including requiring two separate IDA diagnoses separated by 3–12 months to ensure diagnostic accuracy and chronicity, and excluding patients with other forms of anemia that might be misclassified as IDA. Third, because IDA in this study was identified using ICD-10 diagnosis codes, we did not have access to laboratory values such as hemoglobin levels at the time of IDA diagnosis. This limitation prevents us from examining dose–response relationships or identifying particularly high-risk subgroups based on the severity of hemoglobin or iron deficiency. Additionally, we could not assess adherence to iron supplementation or response to treatment, which might modify the thrombotic risk over time. Data on iron preparation use were limited, as only approximately 5% of patients with IDA received iron treatment in our cohort. Consequently, we were unable to conduct meaningful sensitivity analyses comparing VTE risk between treated and untreated patients, and further research is needed to clarify the potential impact of iron therapy. Fourth, our follow-up period of 12 months, while adequate for assessing acute and subacute thrombotic risk, may not capture the long-term effects of IDA on thrombotic risk. Finally, our study’s findings are based on data predominantly from North American populations within the TriNetX network, which may limit generalizability to other regions with different demographic, genetic, or healthcare characteristics. Therefore, validation of these associations in more diverse international cohorts is warranted to confirm the broader applicability of our results.

## 5 Conclusion

This large-scale, multi-institutional matched cohort study provides robust evidence that IDA significantly increases the risk of VTE, with the highest risk occurring during the initial months following diagnosis. Our findings reveal important mechanistic insights, demonstrating that reactive thrombocytosis mediates a substantial portion of the excess DVT risk, while PE risk appears to operate through additional thrombocytosis-independent pathways. This study also highlights the broader clinical impact of IDA, with substantially increased risks of mortality and ICU admission that extend beyond thrombotic complications. Future research should focus on validating these findings in prospective cohorts, investigating optimal prevention strategies for high-risk patients, and elucidating the molecular mechanisms underlying the IDA-VTE relationship. Given the high global prevalence of IDA and the serious nature of venous thromboembolism, these findings represent an important advance in our understanding of preventable thrombotic risk factors, with significant potential for improving patient outcomes through enhanced recognition and management of this common nutritional deficiency.

## Data Availability

The raw data supporting the conclusions of this article will be made available by the authors, without undue reservation.
